# Death in the Digital Age: A Systematic Review of Information and Communication Technologies in End-of-Life Care

**DOI:** 10.1089/jpm.2015.0341

**Published:** 2016-04-01

**Authors:** Kirsten Ostherr, Peter Killoran, Ross Shegog, Eduardo Bruera

**Affiliations:** ^1^ Rice University, Houston, Texas.; ^2^ Department of Anesthesiology, University of Texas Medical School at Houston, Houston, Texas.; ^3^ School of Biomedical Informatics, University of Texas, Houston, Texas.; ^4^ School of Public Health, University of Texas, Houston, Texas.; ^5^ M.D. Anderson Cancer Center, University of Texas, Houston, Texas.

## Abstract

***Background:*** End-of-life (EOL) communication plays a critical role in ensuring that patients receive care concordant with their wishes and experience high quality of life. As the baby boomer population ages, scalable models of end-of-life communication will be needed to ensure that patients receive appropriate care. Information and communication technologies (ICTs) may help address the needs of this generation; however, few resources exist to guide the use of ICTs in EOL care.

***Objective:*** The primary objective was to identify the ICTs being used in EOL communication. The secondary objective was to compare the effectiveness of different ICTs in EOL communication.

***Methods:*** The study was a systematic review, following Preferred Reporting Items for Systematic Reviews and Meta-Analyses (PRISMA) guidelines. We systematically searched seven databases for experimental and observational studies on EOL communication between doctors and patients using ICTs, published in 1997–2013.

***Results:*** The review identified 38 relevant articles. Eleven types of technology were identified: video, website, telephone, videoconferencing, e-mail, telemonitoring, Internet search, compact disc, fax, PalmPilot, and short message service (SMS) text messaging. ICTs were most commonly used to provide information or education, serve as decision aids, promote advance care planning (ACP), and relieve physical symptom distress.

***Conclusions:*** The use of ICTs in EOL care is a small but growing field of research. Additional research is needed to adapt older, analog technologies for use in the digital age. Many of the interventions discussed in this review do not take full advantage of the affordances of mobile, connected health ICTs. The growing evidence base for e-health applications in related fields should guide future interventions in EOL care.

## Introduction

Communication between doctors, patients and families plays a decisive role in ensuring that patients receive end-of-life (EOL) care concordant with their wishes. Yet effective communication between dying patients and health care providers is often lacking,^1–7^ resulting in unwanted intensive interventions, delayed referral to hospice, increased medical costs, feelings of regret among caregivers, and poor quality of life at EOL.^8–25^ Efforts to bring EOL care into accord with patients' wishes through use of advance directives (ADs) and Do Not Resuscitate orders (DNRs) have had limited success, in part because meaningful options are often offered too late,^9,12^ and preferences are rarely documented in the patient's medical record.^26,27^

### The role of information and communication technologies

Programs that link documentation of EOL preferences to an electronic registry demonstrate the potential for new information and communication technologies (ICTs) to make significant improvements to EOL communication.^28–30^ ICTs have begun to transform advance care planning (ACP) by facilitating ease of use, storage, and retrieval of documents;^31^ promoting health literacy; and enabling effective use of palliative care in the EOL decision making context.^32–34^ E-health practices that apply ICTs to the delivery and enhancement of health care services^35,36^ can tailor content to match individual preferences, adapt to diverse cultural norms, and respond to contextually specific cues.^37–39^ These features enable greater efficacy through personalized communication, helping health professionals to reach historically underserved populations more effectively.^40–42^ E-health methods have also improved behavioral outcomes related to important EOL domains such as medication adherence, hospital readmissions, and independent living.^38,43–45^

Consumer-driven, web-based initiatives such as The Conversation Project, Engage with Grace, Five Wishes, and Death over Dinner have encouraged Americans to “have the conversation” at home, “around the kitchen table, not in the ICU” so that family members may be prepared to make decisions before a crisis arises.^25^ These projects further demonstrate the role that ICTs and e-health methods can play in shaping EOL communication outside of the clinical setting; however, little evidence exists of their impact on clinical decision making and patient outcomes.

With the development of new techniques for enhancing and extending doctor-patient communication in the Information Age, researchers need guidance on appropriate uses of technology in EOL care.^48–50^ This systematic review provides a unique and valuable contribution to the research by identifying the uses and evaluating the effects of ICTs in EOL care.

## Methods

This review was conducted following the Preferred Reporting Items for Systematic Reviews and Meta-Analyses (PRISMA) guidelines. ^51^ With the assistance of a health sciences librarian, we searched the literature in seven electronic databases (Medline, PubMed, PsycINFO, Sociological Abstracts, Communication Abstracts, CINAHL, and Embase). The strategy included MeSH headings and keywords related to EOL, doctor-patient communication, and technology. The Medline search strategy can be found in supplementary Table S1. (See online supplementary Table S1 at www.liebertpub.com/jpm and at www.liebertonline.com.) Further detailed search histories are available from the corresponding author upon request.

A randomly selected sample of search results was tested for inter-rater reliability by two independent screeners (K.O. and P.K.) to ensure the validity of the inclusion and exclusion criteria,^52^ resulting in a Cohen's kappa of 0.94. K.O. and P.K. independently screened each title and abstract to identify studies that met inclusion criteria, and disagreements were resolved by consensus based on full-text review.

### Study selection

Inclusion criteria for this systematic review required that studies address EOL communication between doctors and patients; studies focused on removal of life support and/or organ donation that do not include the patient were excluded. The studies had to address EOL communication in patient care; studies that focused solely on training health care providers without implementing the training in a health care setting were excluded. The studies had to include an ICT in the process of communication; studies that did not include any technology were excluded. Studies had to gather quantitative data on efficacy, impact, or effectiveness; studies that were descriptive and/or solely focused on usability or feasibility were excluded. Only articles published in English between 1997, when the Institute of Medicine (IOM) landmark report was published, and 2013 were included in the final review. Only research articles from journals were included; comments, editorials, dissertations, conference proceedings, case reports, etc. were excluded. Cross-sectional, case-control, and cohort studies and clinical trials were included. This study did not require institutional review board approval.

### Data extraction

Full texts of included articles were independently screened by K.O. and P.K. Details of included studies were extracted according to predefined categories. Procedures for coding included methods for assessing risk of bias, based on the Cochrane Collaboration's recommendation in support of using a domain-based evaluation rather than a scale or a checklist.^52^ The quality assessment checklist and summary scores for each included study are available in supplementary Table S2. (See online supplementary Table S2 at www.liebertpub.com/jpm and at www.liebertonline.com.)

## Results

**FIG. 1.**
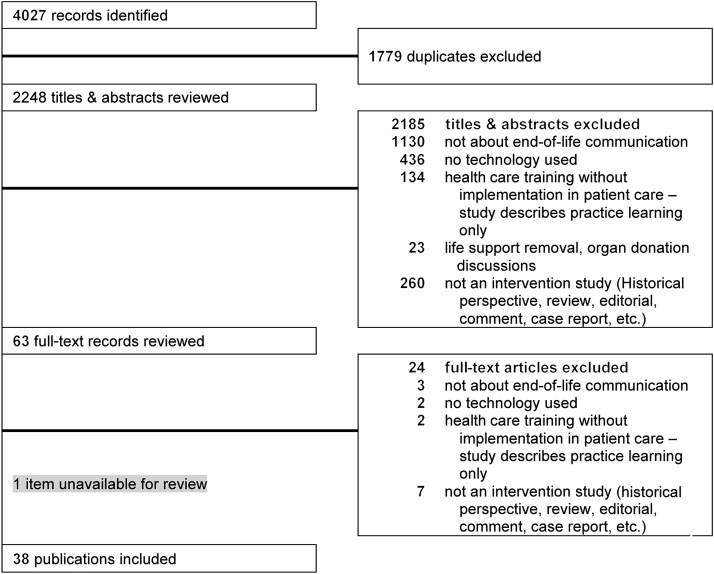
PRISMA flowchart.

Of the initial 2248 articles identified and screened, a total of 38 articles met our inclusion criteria (see Fig. 1 for PRISMA flowchart).^33,34,53–88^ The study populations consisted of two primary patient groups: cancer patients (*n* = 15)^34,53,54,58–62,65,69,73,80,84,85,87^ and noncancer patients (*n* = 23). The noncancer population was subdivided by age, with 29% of the studies^33,55,56,63,64,66,68,72,78,79,81^ focused on populations aged 60 years and older (*n* = 11); 8% of the studies^70,82,83^ focused on populations aged 40 and older (*n* = 3); and 24% of the studies focused on populations defined by other features, including race,^71,86^ primary language,^71^ and referral^57,67,74–77,88^ to palliative care or pain clinic (*n* = 9). Twenty-five^33,34,55,56,58–72,76,78,79,82,83,86^ of the studies were conducted in the United States; three^77,84,85^ in Canada; five in Europe (two^57,80^ in the United Kingdom, one^73^ in Spain, two^53,75^ in The Netherlands); two^87,88^ in Australia; one^81^ in Japan; one^54^ in Korea; and one^74^ in India. All articles were published in the English language (per selection criteria). Eighteen^34,53,61,67–71,75,78,81–88^ were quasi-experimental, pre-post–intervention studies; seventeen^33,54–60,62,64–66,72,73,76,79^ were randomized, controlled trials; two^74,80^ were interrupted time series studies; and one^77^ was a prospective cohort study.

The summary characteristics of these studies are described in Table 1. Details on the bias assessment ratings are included in supplementary Table S2. Complete details on all included studies are available in supplementary Table S3. An explanation of evidence table codes is included in supplementary Table S4. (See online supplementary Tables S2, S3, and S4 at www.liebertpub.com/jpm and at www.liebertonline.com.)

**Table 1.** Summary Overview of Studies (*n* = 38)^a^

*Author, year, country of origin*	*Population*	*Study design*	*Intervention technology*	*Control*	*Results*
*Cancer*					
Epstein et al., 2013, U.S.	Patients with progressive pancreas or hepatobiliary cancer (*n* = 56)	RCT	Educational CPR video about ACP (*n* = 30)	Verbal CPR narrative about ACP (*n* = 26)	**Rates of ACP documentation:** Improved significantly in intervention arm**Knowledge re CPR & ACP:** Increased in both arms**Preferences re CPR:** Decreased in intervention arm but not in control arm **Preferences re mechanical ventilation:** No change in either arm
Gustafson et al., 2013, U.S.	Patients with nonsmall cell lung cancer & their caregivers (*n* = 285 dyads)	RCT	Online support system (Comprehensive Health Enhancement Support System - CHESS) w information, in-site social networking & communication, decision support tools (*n* = 144)	Use of Internet & list of Internet sites about lung cancer (*n* = 141)	**Patient Symptom Distress:** Significantly decreased in intervention arm
Pelayo-Alvarez et al., 2013, Spain	Patients with advanced cancer requiring palliative care (*n* = 169 MDs, 117 patients)	RCT	96 hour online palliative care education program (*n* = 85)	20 hour, face-to-face training course (*n* = 84)	**Patient pain & symptom control:** Significantly improved in intervention arm **Family anxiety:** Significantly lower in intervention arm **Caregiver satisfaction:** No change **Knowledge of MDs:** Significantly increased in intervention arm **Attitude of MDs:** No change
Temel et al., 2013, U.S.	Patients with incurable lung cancer (*n* = 181)	Pre-post	E-mail prompt timed to treatment events (*n* = 98)	Usual care (hist. controls) (*n* = 83)	**Clinician documentation of code status in EHR:** Significantly increased in intervention arm **Mean time to code status documentation in EHR:** Significantly decreased in intervention arm
Vogel et al., 2013, U.S.	Women with ovarian cancer (*n* = 35)	RCT	Prototype website to help patients monitor distress, record questions for providers, access information, set goals, tailored to disease stage & learning style (*n* = 20)	Control website (*n* = 15)	**Completion of AHD:** No change **Palliative care consultation:** No change **Decisional conflict:** Improved in intervention arm
Volandes et al., 2013, U.S.	Patients with advanced cancer (*n* = 150)	RCT	Video decision support tool depicting patient on ventilator & CPR performed on simulated patient (*n* = 70)	Verbal narrative describing CPR (*n* = 80)	**Preference for CPR:** Significantly lower in intervention arm **Knowledge of CPR:** Significantly increased in intervention arm
Watanabe et al., 2013, Canada	Rural patients with advanced cancer (*n* = 44)	Pre-post	Videoconferencing for patients at rural health care facility (*n* = 44)	Reflexive	**Patient anxiety:** Significantly improved **Patient appetite:** Significantly improved **Cost in time, distance traveled, & dollars:** Significantly reduced
Volandes et al., 2012, U.S.	Patients with advanced cancer (*n* = 80)	Pre-post	Educational video (*n* = 80)	Reflexive	**Goals of care preference:** No change **CPR preference:** Decreased **Knowledge re goals of care:** Increased
Green, Levi, 2011, U.S.	2nd year medical students (*n* = 121) & patients with cancer (*n* = 121)	Pre-post	Computer-based multimedia decision aid designed to help patients w ACP (*n* = 60)	Standard advance directive form (*n* = 61)	**Patient satisfaction w ACP methods:** Significantly higher in intervention arm
Uitdehaag et al., 2011, Netherlands	Patients w new diagnosis of incurable esophageal or head & neck cancer (*n* = 17)	Pre-post	Audiorecording (CD) of diagnostic consultation, delivery of diagnosis, & discussion of palliative care (*n* = 10)	Consultation without audiorecording (*n* = 7)	**Patient QOL:** Decreased in intervention arm **Patient communication:** Improved in intervention arm
Yun et al., 2011, Korea	Caregivers of terminally ill patients w cancer & their patients (*n* = 444)	RCT	Decision aid (video & companion workbook) showing how to discuss terminal prognosis w patient (*n* = 216)	Decision aid (video & companion workbook) showing pain management techniques (*n* = 228)	**Decision to communicate:** No change **Caregivers' decisional conflict:** Improved significantly more in intervention arm than in control arm **Depression:** Improved significantly more in experimental arm than in control arm
Capewell et al., 2010, U.K.	Palliative care cancer patients (*n* = 15)	Interrupted time series	6 minute video on aspects of cancer pain & use of strong opioids (*n* = 15)	Reflexive	**Patient pain scores:** Significantly improved
El-Jawahri et al., 2010, U.S.	Patients with malignant glioma (*n* = 50)	RCT	Video depicting life-prolonging, basic, & comfort care (*n* = 23)	Verbal narrative on goals-of-care options at EOL (*n* = 27)	**Preferences for EOL care:** None preferred life-prolonging care; significantly fewer preferred basic care; significantly more preferred comfort care in intervention arm **Uncertainty re decision making:** Significantly higher in intervention arm
Duggleby et al., 2007, Canada	Terminally ill cancer patients age ≥60 years (*n* = 60)	Pre-post	Research-based video & choice of hope activities w instruction by trained RNs (*n* = 30)	Usual care (*n* = 30)	**Patient hope:** Significantly higher in intervention arm **Patient QOL:** Significantly higher in intervention arm
*Nonspecific diagnosis, age ≥65*					
Volandes et al., 2012, U.S.	Patients age ≥65 years admitted to 2 skilled nursing facilities (*n* = 101)	RCT	Video describing goals-of-care options (*n* = 50)	Verbal narrative (*n* = 51)	**Patient preferences for comfort care:** Significantly higher in intervention arm
Volandes et al., 2011, U.S.	Subjects age ≥65 years at rural primary care clinic (*n* = 76)	RCT	Verbal description of advanced dementia & goals of care & video decision aid (*n* = 33)	Verbal description of advanced dementia & goals of care (*n* = 43)	**Patient preferences for comfort care:** Slightly higher in intervention arm
Hamlet et al., 2010, U.S.	Medicare beneficiaries who need EOL care planning (*n* = 4742)	RCT	Telephone-based education & counseling about AD, palliative versus aggressive care, hospice enrollment; facilitation of interactions with physicians & hospice agencies; referrals to hospice when appropriate; caregiver support (*n* = 3112)	Usual care (*n* = 1630)	**Election of less-aggressive care:** Significantly higher in intervention arm **Increased rate of hospice enrollment:** No change **Duration of hospice care prior to death:** No change
Matsui, 2010, Japan	Japanese adults age ≥65 years (*n* = 121)	Pre-post	Video & lecture using handout (*n* = 55)	Handout only (*n* = 57)	**Attitudes towards ADs:** Improved slightly in intervention arm **Preference for life-sustaining tx by artificial nutrition:** Decreased significantly in intervention arm **Discussion of EOL preferences w family or HCP:** Increased significantly in intervention arm
Volandes et al., 2009, U.S.	Older people age >65 years living in the community (*n* = 200)	RCT	Verbal narrative describing advanced dementia, 2 minute video depicting patient w advanced dementia (*n* = 94)	Verbal description alone (*n* = 106)	**Election of less aggressive care:** Significantly higher in intervention arm
Volandes et al., 2009, U.S.	Community-dwelling subjects age ≥65 years & surrogates (*n* = 28; 14 dyads)	RCT	Verbal narrative & 2 minute video decision support tool depicting patient with advanced dementia (*n* = 16; 8 dyads)	Verbal narrative describing advanced dementia (*n* = 12; 6 dyads)	**Patient preferences for comfort care:** Significantly higher in intervention arm **Concordance of surrogate prediction & patient preferences:** Significantly higher in intervention arm
Clarke, et al., 2005, U.S.	Senior citizens in community organizations (*n* = 944)	Pre-post	Mailed flyer offered home-based critical care & AD guide, & individual telephone counseling (*n* = 839)	Mailed flyer only (*n* = 105)	**Completion of AD:** Slightly higher in intervention arm
Brown et al., 1999, U.S.	Patients age ≥75 years who used Franklin Medical Office (*n* = 1247)	RCT	Videotape w illustrated pamphlet, AD forms & guide (*n* = 619)	Illustrated pamphlet, AD forms & guide (*n* = 628)	**Placement of AD in medical record:** Increased in both groups, no significant difference between groups
Yamada et al., 1999, U.S.	Veterans age ≥70 years, deemed able to make medical care decisions (*n* = 117)	RCT	Two handouts on AD & CPR, & 10 minute video about ADs (*n* = 62)	Handout explaining ADs but not CPR (*n* = 55)	**Knowledge about AD:** Significantly higher in intervention arm **Discussion of CPR or AD w HCP:** No change
*Nonspecific diagnosis, other*					
Kannan, Kamalini, 2013, India	Palliative care outpatients (*n* = 60)	Interrupted time series	SMS text messaging between MDs & patients to manage analgesic adherence & titration for pain relief	Historical data	**Doctor-patient communication re pain relief:** Significantly increased
Sudore et al., 2013, U.S.	Racially & ethnically diverse, low-income adults age ≥60 years (*n* = 43)	Pre-post	Prototype website guided patients to identify life goals & preferences for medical care & to communicate preferences to surrogate decision makers & physicians	Reflexive	**Engagement in ACP behaviors:** Significantly increased
Takahashi et al., 2012, U.S.	Patients age >60 years w chronic health problems at high risk of hospitalization & ED visits (*n* = 205)	RCT	Patients relayed biometric & clinical information by telemonitoring to RN. Medical care team communicated with patient via phone or videoconferencing as needed (*n* = 102)	Usual care (*n* = 103)	**Hospice enrollment:** Increased in intervention arm **Mean number of days in hospice:** Decreased in intervention arm **Time to hospice referral:** No difference
Deep, et al., 2010, U.S.	Patients age >40 years scheduled to see general internist (*n* = 120)	Pre-post	Video of patient w advanced dementia	Verbal description of advanced dementia	**Patient preferences for comfort care:** Significantly increased
Kersholt et al., 2009, Netherlands	Members of choirs & music associations (*n* = 183)	Pre-post	Direct assessment (*n* = 66), text stories (*n* = 63), & video (*n* = 52) to elicit preferences re: location of death at home vs hospice vs nursing home	Reflexive	**Patient preference for home death:** Decreased w text & video **Patient preference for hospice:** Increased w text, decreased w video **Patient preference for nursing home:** Increased w video
Volandes et al., 2009, U.S.	Adult patients age ≥40 years scheduled to see general internist (*n* = 146)	Pre-post	Video decision aid showing patient w salient features of advanced dementia	Verbal description of advanced dementia	**Patient uncertainty re EOL preferences:** Decreased
Schofield et al., 2008, Australia	Patients scheduled to receive first ever chemotherapy treatment (*n* = 100)	Pre-post	Educational video on preparing for chemotherapy & self-management of 8 common side effects (*n* = 50)	Usual care (historical controls) (*n* = 50)	**Patient self-efficacy for seeking social support:** Significantly increased **Patient satisfaction w information re side effects:** Significantly increased
Volandes et al., 2008, U.S.	Spanish-speaking patient of primary care doctors (*n* = 104)	Pre-post	Two-minute video decision support tool depicting patient with salient features of advanced dementia	Reflexive	**Patient preferences for comfort care:** Significantly increased
Volandes et al., 2008, U.S.	White & African American patients presenting to primary care doctors (*n* = 144)	Pre-post	Video decision support tool depicting patient with salient features of advanced dementia to help patients overcome low health literacy barriers	Verbal description of advanced dementia	**Low health literacy patient preferences for aggressive care:** Significantly decreased **Low health literacy patient preferences for comfort care:**Significantly increased
Penrod et al., 2007, U.S.	Patients at 5 acute care & 3 nursing home sites (*n* = 3557)	Pre-post	Web-based palliative care report card implemented on network Intranet	Historical data	**Number of PC consults:** Significantly increased **Percentage of inpatient deaths w PC consultation:** Significantly increased **Average days betw initial PC consultation & death:** Significantly increased
Volandes et al., 2007, U.S.	Patients age >40 years scheduled to see general internist (*n* = 120)	Pre-post	Video decision support tool depicting patient with advanced dementia	Verbal description of patient with advanced dementia	**Patient preferences for comfort care:** Significantly increased
Brumley, et al., 2006, Australia	All adult patients admitted to domiciliary palliative hospice care (*n* = NR)	Pre-post	One-page information sheet (on MS Word program) updated daily for each pt on computers, faxed to GPs, & downloaded to nurses' PalmPilots	Reflexive	**Patient outcomes:** Improved
Hanks et al., 2002, U.K.	New inpatient referrals to the palliative care team (PCT) (*n* = 261)	RCT	Telephone & in-person advice & support by PCT (*n* = 175)	Limited telephone advice only by PCT (*n* = 86)	**Symptom control:** Significantly improved in intervention arm **HRQoL:** Significantly improved in intervention arm **Mood:** Significantly improved in intervention arm **Emotional bother:** Significantly improved in intervention arm
Gammaitoni et al., 2000, U.S.	Patients enrolled at university pain clinic (*n* = 74)	RCT	Telephone-based pharmaceutical palliative care program w specialized prescription services tailored to needs of pain medicine clinical practice (*n* = 38)	Usual care (*n* = 36)	**Patient access to medication:** Increased **Efficient processing of prescriptions:** Increased **Patient experience of stigmatization:** Decreased
Ho, et al., 2000, Canada	Patients with HIV/AIDS (*n* = 140)	Prospective cohort study	AD documents, educational video, & three individual face-to-face counseling sessions	Reflexive	**AD completion rates:** Significantly increased **Legal validity of completed ADs:** Significantly decreased **Patient satisfaction w health care:** Decreased

^a^ See Table S3 for detailed information on all included studies and Table S4 for a complete explanation of evidence table codes.

NR, none reported.

### Types of technology used

Eleven types of technology were used in the included studies, with some studies employing more than one type. Of these, 55% (*n* = 22) used video^33,34,54,56,59,62,64–66,70–72,75,77,79–84,86,87^ as the intervention technology; 15% (*n* = 6) developed a prototype website;^58,60,67,69,73,78^ 10% (*n* = 4) used a telephone;^55,57,68,76^ and the remaining technologies—videoconferencing,^85^ e-mail prompt,^61^ telemonitoring,^63^ Internet search^60^ (without developing prototype website), compact disc,^53^ fax,^88^ PalmPilot, ^88^ and SMS text messaging^74^ —were used once each.

### Technology by date of study

**FIG. 2.**
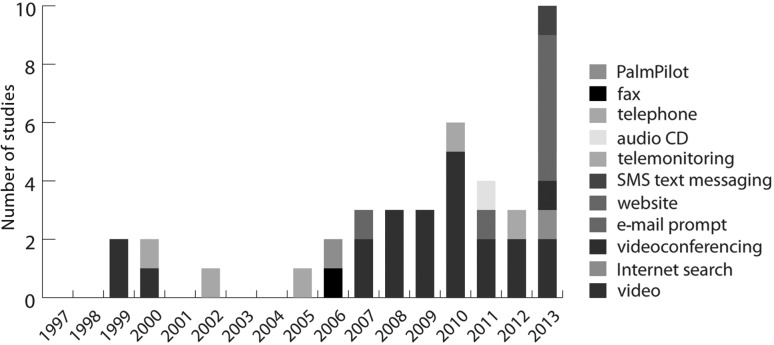
Technology by date of study.

Research on ICTs in EOL care has grown significantly in recent years (see Fig. 2). Between 1997–2009, 16 studies^56,57,66–68,71,72,75–77,79,83,84,86–88^ that met our inclusion criteria were published in this field, averaging less than one study per year. In contrast, 22 studies^33,34,53–55,58–65,69,70,73,74,78,80–82,85^ (58% of included studies) were published between 2010–2013, averaging five studies per year, with nine of the studies^58–62,73,74,78,85^ included in this review (24%) published in 2013 alone.

### Purpose of technology

**FIG. 3.**
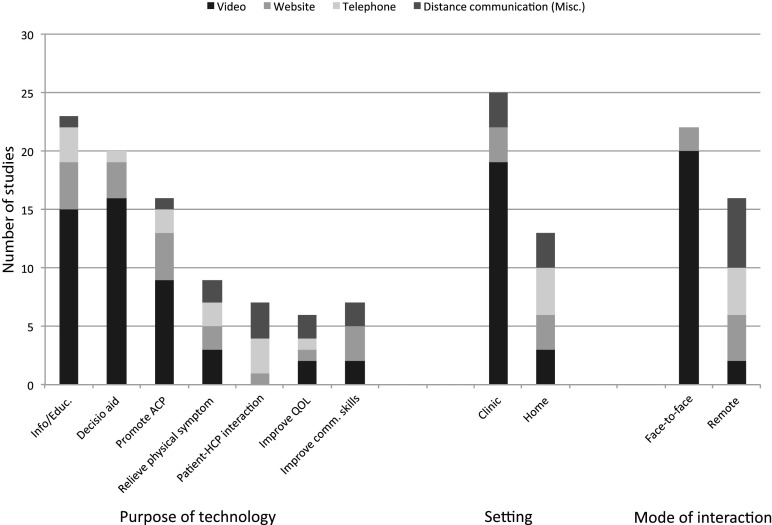
Purpose of technology (by type of ICT).

Seven purposes of ICT use in EOL care were identified. The most common purposes for using technology in these interventions were to provide information/education and to serve as decision aids, followed by promoting ACP and/or documenting a patient's code status, and relieving physical symptom distress. See Figure 3 on the purposes of technology use, setting (clinic or home), and mode of interaction (remote or face-to-face).

On average, each intervention used ICTs for at least two purposes, with 61% of studies (*n* = 23) using technology to provide information/education, and 53% of studies (*n* = 20) using technology as a decision aid. Approximately 24% of the studies in this review (*n* = 9) used ICTs to relieve physical symptom distress. While this sample size is relatively small, the use of technology to enable remote interventions for managing patient pain is extremely promising for delivering improved health outcomes, quality of life, and cost savings for patients at the EOL.

## Discussion

The aim of this study was to review existing studies describing the use of ICTs in EOL care for communication between doctors, patients, and family members. The results show that ICT use in EOL care is an emergent and expanding area of research, with a variety of ICT tools undergoing rigorous evaluation. Although the field is young, almost half (45%) of the included studies were randomized, controlled trials—the gold standard in evidence-based medicine—and therefore, the evidence base for the field shows promising signs of maturation. However, many of the studies compared the effectiveness of the ICT intervention to “usual care,” with fewer studies comparing effectiveness among different ICTs. Given the widespread recognition from the IOM and the research cited here that “usual care” for patients at the EOL is inadequate, this measure of comparison does not provide results that are as meaningful as they should be.

An additional feature of the relative immaturity of this field is its lack of unbiased research. For bias risk criteria, the most problematic categories were blinding of participants and personnel, allocation concealment, and blinding of outcome assessment. Many of these biases were due to the inherent limitations of small sample sizes and the use of a pre-post intervention study design with no control group. Finally, the sensitive nature of conducting research on patients nearing EOL poses both ethical and logistical challenges that require researchers to develop innovative techniques that do not always conform to the scientific gold standard of the double-blind, randomized, controlled trial. This field of research may require ongoing development of innovative, field-specific standards for research validity as it matures.

### Web-based interventions

While the older technologies identified in this review, such as telephone and video, continue to be studied, the data indicate a trend toward increasing use of Internet-based interventions. Notably, four of the six studies in this review that developed prototype websites for health intervention were published in 2013. This shift is likely due to technological and infrastructure enhancements that have enabled access to high-speed broadband Internet across most of the United States, Canada, and western European countries. The recent studies using videoconferencing and e-mail prompts also reflect this shift toward delivering health interventions through networked ICTs. (See supplementary Table S5 for links to relevant web resources.) (See online supplementary Table S5 at www.liebertpub.com/jpm and at www.liebertonline.com.)

### Video

With over half of the included studies (*n* = 22) using video as their intervention technology, the evidence base for the utility of this type of ICT in EOL communication is strong. In particular, numerous studies demonstrated the efficacy of video as a decision support tool in ACP. These results are not surprising, considering that the evidence base for video-based telehealth dates back to the 1950s.^89,90^ In addition, ICTs including video have been used for information/education and as decision aids in behavioral health interventions for other fields, where a variety of media technologies have been previously validated for these purposes.^38,43–45,91,92^ However, none of the video interventions employed mobile platforms to deliver the video, nor did they engage patients via popular video sites on the Internet. Interventions using video as a decision support tool in EOL care should begin to include mobile applications of video.

### Telemedicine

None of the telephone-based studies included in this review used cellular phones or smartphones. However, the same principles concerning effects of face-to-face versus remote care would likely apply in both settings. Moreover, the evidence base for mHealth interventions using mobile phones for other health problems may provide valuable models for applications in EOL care. Evidence from related connected health fields such as remote ICU monitoring show promise and should guide future interventions in connected EOL care.^44,93^

Several studies using new communication tools and techniques such as Skype,^94^ Twitter,^95^ and blogging^96^ were excluded on the basis of their study design, but suggest areas for further development of research to compare their effectiveness with analogous ICTs, such as hospital-based videoconferencing and e-mail. When possible, ICTs should be compared to one another, rather than solely to usual care.

### ICTs for cancer population

Research on the use of ICTs in EOL care has developed most robustly among cancer researchers, with almost half the studies in this review focusing on cancer patients. It is possible that this emphasis is due to the better-known trajectory of certain types of terminal cancer, which may make the EOL stage of cancer more amenable to study than many other diseases. In addition, because cancer strikes across socioeconomic classes, it may be more feasible to pilot test new forms of health interventions that depend on ICT use enabled by high-speed Internet connectivity and/or that depend on study subjects willing to engage with new technologies.

### Limitations

A limitation of this review is the small sample size. Although the initial search captured over 2000 results, after applying the inclusion and exclusion criteria, the sample was reduced to only 38 studies. The widely heterogeneous results in this sample made comparison of effectiveness impossible; therefore the second objective of the study was not met. This review was limited to articles published in the English language, and therefore it may have missed significant research and trends taking place in other languages. In addition, the scope of this systematic review is broad, and the study covers diverse types of interventions. As a result, we were unable to calculate effect sizes. Finally, this review studies the use of ICTs in isolation from other dimensions of communication in patient care, both inside and outside of the health care setting. While the role of ICTs in EOL care shows significant promise, the use of technology will be but one among many different forms of communication that occur between the patient, family members, caregivers, and health care providers. The use of technologies in these settings will be best understood when studied in relation to other aspects of human communication.

## Conclusions

Based on this systematic review of the literature on ICT use in EOL care, the authors have identified several opportunities for further research. First, future research should take advantage of the affordances of mobile, connected, health ICTs. Second, the proven value of video in helping patients clarify their treatment preferences should encourage more providers to experiment with this medium using mobile devices. Third, research is needed to help health care providers determine when face-to-face communication with patients is necessary, and when remote communication will achieve comparable objectives. If the results of such comparisons became generalizable, that would enable more rapid uptake of new technologies as they emerge.

Scalable innovations are sorely needed to improve quality of life at EOL while reducing the costs of care. ^97^ Although emerging technologies are often associated with younger rather than older users, research shows that more and more aging Americans are using the Internet and connected health technologies, and this generational change will only increase as baby boomers grow old.^3^ As increasing numbers of Americans approach their final days with their laptops, smartphones, and tablets by their side, the use of ICTs to help them manage their health will become unavoidable. To ensure that mobile, networked ICTs are used effectively to optimize EOL care, it is essential that forward-looking research builds on the existing evidence base and continues to explore new techniques for delivering health care in the 21st century.

## Supplementary Material

Supplemental data

Supplemental data

Supplemental data

Supplemental data

Supplemental data
