# Supplementary material for: Death in the Digital Age: A Systematic Review of Information and Communication Technologies in End-of-Life Care
Source: J Palliat Med. 2016 Apr 1;19(4):408–20. doi: 10.1089/jpm.2015.0341 (PMC4827321; doi:10.1089/jpm.2015.0341)
Supplement: Supplemental data [file Supp_Table1.pdf]

Supplementary Data

Supplementary Table S1. Ovid Medline Search Strategy

**Provider/Interface**	Ovid
**Database**	Medline®
**Date searched**	1/27/2014
**Database update**	1996 to January week 3 2014; in-process & other nonindexed citations January 24, 2014
**Search developer(s)**	Helena M. VonVille; Kirsten Ostherr
**Language**	English
**Date range**	1997–2013
**Search filter**	libguides.sph.uth.tmc.edu/ovid_medline_filters
1	advance care planning/
2	palliative care/or terminal care/or hospice care/
3	(advance care or palliative or hospice or ''end of life'' or eol or terminal care).ti,ab.
4	1 or 2 or 3
5	audiovisual aids/or blogging/or books, illustrated/or books/or cd-rom/or communications media/ or compact disks/or computer-assisted instruction/or computers, handheld/or electronic mail/ or internet/or mass media/or motion pictures as topic/or multimedia/or optical storage devices/ or pamphlets/or remote consultation/or social media/or tape recording/or teaching materials/or telecommunications/or telemedicine/or telephone/or television/or text messaging/or video recording/or videoconferencing/or videodisc recording/or videotape recording/
6	(advergam* or advertis* or app or apps or audiovisual or automated voice or avatar or blog or blogged or blogging or cell phone* or chat room* or commercial* or computer game* or cyberspace or ddr or dvd or dvds or ''dvd's'' or dvds or educational entertainment or edutainment or email* or facebook or flickr or foursquare or friending or game* or gaming or Google or ''Google+'' or Googling or Googleplus or hand-held or handheld* or ''health 2.0'' or instagram or instant messag* or internet or ipad or iphone or ipod or LinkedIn or listserv* or mass media or messag* or microblog* or mobile phone* or myspace or ''new media'' or ''new technology'' or ''new technologies'' or nintendo or online or orkut or pinterest or pod cast or pod casts or podcast* or psa or psas or ''psa's'' or public service announcement* or push* content or Reddit or ''short message service'' or skype or small media or smart phone* or smartphone* or sms or social media or social network* or social web* or ''tablet computer'' or telemedicine or telephon* or television* or text messag* or texting or toolkit* or tumblr or tweet* or twitter or video blog or video game* or video log or video* or vine or ''virtual reality'' or vlog* or ''web 2.0'' or web page* or web site* or web-based learning or webinar* or weblog* or website* or wii or wiki* or wireless or world wide web or xbox or x-box or you tube or youtube).ti,ab.
7	5 or 6
8	4 and 7
9	(''clinical trial'' or ''clinical trial, phase i'' or ''clinical trial, phase ii'' or clinical trial, phase iii or clinical trial, phase iv or controlled clinical trial or ''multicenter study'' or ''randomized controlled trial'').pt. or double-blind method/or clinical trials as topic/or clinical trials, phase i as topic/or clinical trials, phase ii as topic/or clinical trials, phase iii as topic/or clinical trials, phase iv as topic/or controlled clinical trials as topic/or randomized controlled trials as topic/or early termination of clinical trials as topic/or multicenter studies as topic/or ((randomi?ed adj7 trial*) or (controlled adj3 trial*) or (clinical adj2 trial*) or ((single or doubl* or tripl* or treb*) and (blind* or mask*))).ti,ab.
10	evaluation studies/or evaluation studies as topic/or program evaluation/or validation studies as topic/or intervention studies/or ((pre- adj5 post-) or (pretest adj5 posttest) or (program* adj6 evaluat*)).ti,ab. or (effectiveness or intervention*).ti,ab.
11	(((''semi-structured'' or semistructured or unstructured or informal or ''in-depth'' or indepth or ''face-to-face'' or structured or guide) adj3 (interview* or discussion* or questionnaire*)) or (focus group* or qualitative or ethnograph* or fieldwork or ''field work'' or ''key informant'')).ti,ab. or interviews as topic/or focus groups/or narration/or qualitative research/
12	questionnaires/or (questionnaire* or survey*).ti,ab.
13	data analysis.ti,ab.
14	9 or 10 or 11 or 12 or 13
15	8 and 14
16	limit 15 to yr = ''1997 – 2013''
17	limit 16 to english language

ab, abstract; pt, patient; ti, title.
