# Supplementary material for: Death in the Digital Age: A Systematic Review of Information and Communication Technologies in End-of-Life Care
Source: J Palliat Med. 2016 Apr 1;19(4):408–20. doi: 10.1089/jpm.2015.0341 (PMC4827321; doi:10.1089/jpm.2015.0341)
Supplement: Supplemental data [file Supp_Table2.pdf]

Supplementary Table S2. Risk of Bias Rating (Ordered by Sum Total Score)

Author, pub. date [ref ID]	*Random sequence generation*	*Allocation concealment*	*Blinding of participants and personnel*	*Blinding of outcome assessment*	*Incomplete outcome data*	*Selective reporting*	*Other sources of bias*	*Total*
Uitdehaag et al., 2012 [186]	Y	Y	N	Y	N	Y	N (S)	**4/7**
Yun et al., 2011 [208]	Y	Y	N	N	Y	Y	N (NRS)	**4/7**
Hamlet et al., 2010 [397]	Y	N	N	N	Y	Y	Y	**4/7**
Volandes et al., 2009 [489]	Y	Y	N	N	Y	Y	N (NRS, OC)	**4/7**
Hanks et al., 2002 [953]	Y	Y	N	N	Y	Y	N (NRS, S)	**4/7**
Vogel et al., 2013 [4]	Y	N	N	N	Y	Y	N (NRS)	**3/7**
Epstein et al., 2013 [32]	Y	N	N	N	Y	Y	N (NRS)	**3/7**
Gustafson et al., 2013 [39]	Y	N	N	N	Y	Y	N (OC)	**3/7**
Temel et al., 2013 [61]	N	N	N	N	Y	Y	Y	**3/7**
Volandes et al., 2013 [68]	Y	N	N	N	Y	Y	N (M, NVT)	**3/7**
Takahashi et al., 2012 [88]	Y	N	N	Y	N	Y	N (S)	**3/7**
Volandes et al., 2012 [143]	Y	N	N	N	Y	Y	N (NRS)	**3/7**
Volandes et al., 2011 [309]	Y	N	N	N	Y	Y	N (S, NRS)	**3/7**
El-Jawahri et al., 2010 [444]	Y	N	N	N	Y	Y	N (S, NGS)	**3/7**
Volandes et al., 2009 [464]	Y	N	N	N	Y	Y	N (S)	**3/7**
Penrod et al., 2007 [657]	N	N	N	N	Y	Y	Y	**3/7**
Clarke et al., 2005 [802]	Y	N	N	N	Y	Y	N (NC)	**3/7**
Green, Levi, 2011 [303]	N	N	N	N	Y	Y	N (M)	**2/7**
Deep et al., 2010 [348]	N	N	N	N	Y	Y	N (NRS, NC)	**2/7**
Volandes et al., 2008 [577]	N	N	N	N	Y	Y	N (M)	**2/7**
Yamada et al., 1999 [1059]	Y	N	N	N	Y	Y	N (S)	**3/7**
Pelayo-Alvarez et al., 2013 [1151]	Y	N	N	N	Y	Y	N (M)	**3/7**
Kannan, Kamalini, 2013 [1189]	N	N	N	N	Y	Y	N (NRS, NC)	**2/7**
Kersholt et al., 2009 [3539]	N	N	N	N	Y	Y	N (NRS, NC)	**2/7**
Gammaitoni et al., 2000 [1224]	Y	N	N	N	N	Y	N (S)	**2/7**
Ho et al., 2000 [1056]	N	N	N	N	Y	Y	N (OC)	**2/7**
Sudore et al., 2013 [1253]	N	N	N	N	Y	Y	N (S, NRS, M)	**2/7**
Brown et al., 1999 [3979]	Y	N	N	N	N	Y	N (M)	**2/7**
Volandes et al., 2012 [121]	N	N	N	N	Y	Y	N (NRS)	**2/7**
Capewell et al., 2010 [353]	N	N	N	N	Y	Y	N (S)	**2/7**

(*continued*)

Supplementary Table S2. (Continued)

Author, pub. date [ref ID]	*Random sequence generation*	*Allocation concealment*	*Blinding of participants and personnel*	*Blinding of outcome assessment*	*Incomplete outcome data*	*Selective reporting*	*Other sources of bias*	*Total*
Matsui, 2010 [400]	N	N	N	N	Y	Y	N (S)	**2/7**
Volandes et al., 2010 [439]	N	N	N	N	Y	Y	N (M)	**2/7**
Volandes et al., 2007 [677]	N	N	N	N	Y	Y	N (M)	**2/7**
Duggleby et al., 2007 [686]	Y	N	N	N	N	Y	N (S)	**2/7**
Watanabe et al., 2013 [57]	N	N	N	N	N	Y	N (NC)	**1/7**
Volandes et al., 2008 [576]	N	N	N	N	N	Y	N (M)	**1/7**
Schofield et al., 2008 [626]	N	N	N	N	N	Y	N (S, M)	**1/7**
Brumley et al., 2006 [720]	N	N	N	N	N	N	N (M)	**0/7**
**Total**	**20/38**	**4/38**	**0/38**	**2/38**	**29/38**	**37/38**	**3/38**	

M, multiple other sources of bias; NA, not available; NC, no comparator; NRS, nonrepresentative sample; NVT, nonvalidated measurement tool; OC, other confounders; S, small sample size.

Ref ID for internal documentation available on request from corresponding author.
