# Supplementary material for: Death in the Digital Age: A Systematic Review of Information and Communication Technologies in End-of-Life Care
Source: J Palliat Med. 2016 Apr 1;19(4):408–20. doi: 10.1089/jpm.2015.0341 (PMC4827321; doi:10.1089/jpm.2015.0341)
Supplement: Supplemental data [file Supp_Table3.pdf]

Supplementary Table S3. Detailed Overview of Studies Organized by Patient Population and Date of Publication ($N = 38$)[a]

Author, year, and country of origin	*Population*	*Study design*	*Intervention*	*Comparison*	*Methods*	*Outcomes*	*Results*
Cancer							
Epstein et al., 2013, U.S.	Patients with progressive pancreas or hepatobiliary cancer ($n = 56$)	RCT	Educational video ($n = 30$)	Verbal narrative ($n = 26$)	Patients were randomized to educational CPR video or similar CPR narrative about ACP.	1a: Difference in ACP documentation one month posttest between arms.<br>2a: Study impressions; pre- & postintervention knowledge of, preferences for CPR, mechanical ventilation; longitudinal patient outcomes.	Rates of ACP documentation: 40% in video arm (12/30) & 15% in narrative arm (4/26), OR = 3.6 [95% CI: 0.9–18.0], $p = 0.07$. Postintervention knowledge higher in both arms. Posttest preferences for CPR had changed in video arm but not in narrative arm. Preferences re: mechanical ventilation did not change in either arm.
Gustafson et al., 2013, U.S.	Patients with nonsmall cell lung cancer & their caregivers ($n = 285$ dyads)	RCT	Online support system (CHESS) ($n = 144$)	Use of the Internet & a list of Internet sites about lung cancer ($n = 141$)	Caregiver-patient dyads received standard care plus training on & access to Internet & list of sites about lung cancer or CHESS (information, in-site social networking & communication, decision-support tools).	Patient symptom distress.	Caregivers in CHESS arm consistently reported lower patient physical symptom distress than caregivers in Internet arm. Significant differences were observed at 4 months ($P = 0.031$; Cohen $d = 0.42$) & at 6 months ($P = 0.004$; $d = 0.61$). Similar but marginally significant effects were observed at 2 months ($P = 0.051$; $d = 0.39$) & at 8 months ($P = 0.061$; $d = 0.43$).
Pelayo-Alvarez et al., 2013, Spain	Patients with advanced cancer requiring palliative care ($n = 169$ MDs, 117 patients)	RCT	Online palliative care education ($n = 85$)	Traditional training ($n = 84$)	Intervention group had access to 96 hour online program for PC self-training. Control group could voluntarily receive face-to-face, 20 hour PC training course. Consecutive patients with advanced cancer requiring PC were included.	Patient symptom control & QOL; caregiver satisfaction; knowledge/attitude of MDs.	Intervention group had reduced scores for pain, symptoms, & family anxiety. Global RSCL scale showed a difference between groups. Caregiver satisfaction was comparable between groups. MDs in intervention group significantly increased knowledge without any differences in attitude. Online training was completed by 86.6% in intervention group; 13.4% in control group accessed traditional training.

(*continued*)

Supplementary Table S3. (Continued)

Author, year, and country of origin	*Population*	*Study design*	*Intervention*	*Comparison*	*Methods*	*Outcomes*	*Results*
Temel et al., 2013, U.S.	Patients with incurable lung cancer ($n=181$)	Pre-post	EPP ($n=98$)	Usual care (hist. controls) ($n=83$)	Outpatient oncology clinical team received e-mail prompts timed to treatment events.	Clinician documentation of code status in the HER.	At 1 year follow-up, 33.7% ($n=33/98$) of EPPs had code status documented in outpatient EHR compared with 14.5% ($n=12/83$) of historical controls ($P=0.003$). Mean time to code status documentation was significantly shorter in EPPs (8.6 months [95% CI, 7.6–9.5]) compared with controls (10.5 months [95% CI, 9.8–11.3]; $P=0.004$).
Vogel et al., 2013, U.S.	Women with ovarian cancer ($n=35$)	RCT	Prototype website ($n=20$)	Control website ($n=15$)	Prototype website developed to help patients monitor distress, record questions to ask providers, access information, set goals, tailored to disease stage & learning style.	Completion of advanced health care directive & palliative care consultation.	No statistical differences in completion of AHD ($p=0.220$) or palliative care consultation ($p=0.440$) between intervention & controls. However, intervention group showed evidence of moving toward decision making regarding AHD & palliative care & lower decisional conflict. Compared to controls, intervention group was highly satisfied with amount ($p=0.054$) & quality ($p=0.119$) of information; & when they accessed the website they used it longer ($p=0.049$).
Volandes et al., 2013, U.S.	Patients with advanced cancer ($n=150$)	RCT	Video decision support tool ($n=70$)	Verbal narrative ($n=80$)	Control arm listened to verbal narrative describing CPR & likelihood of successful resuscitation. Intervention arm listened to same narrative & viewed 3 minute video depicting patient on ventilator & CPR performed on simulated patient.	1a: Participants' preference for or against CPR measured immediately after exposure to either modality.<br>2a: Participants' knowledge of CPR & comfort with video.	Control arm: 38 participants (48%) wanted CPR, 41 (51%) wanted no CPR, 1 (1%) was uncertain. Intervention arm: 14 participants (20%) wanted CPR, 55 (79%) wanted no CPR, 1 (1%) was uncertain (unadjusted odds ratio, 3.5; 95% CI, 1.7–7.2; $P=0.001$). Mean knowledge scores higher in intervention arm than in control arm (3.3 ± 1.0 v 2.6 ± 1.3, respectively; $P=0.001$); 65 participants (93%) in the intervention arm were comfortable watching the video.

(*continued*)

Supplementary Table S3. (Continued)

Author, year, and country of origin	*Population*	*Study design*	*Intervention*	*Comparison*	*Methods*	*Outcomes*	*Results*
Watanabe et al., 2013, Canada	Rural patients with advanced cancer ($n=44$)	Pre-post	Videoconferencing ($n=44$)	Reflexive	Patients presented to rural health care facility for scheduled videoconference.	Symptom, cost, & satisfaction outcomes.	Statistically significant improvement in mean ESAS scores for anxiety ($p<0.01$) & appetite ($p=0.03$) at first follow-up. Average per visit savings for patients seen by telehealth versus attending clinic were 471.13 km, 7.96 hours, and Cdn $192.71, respectively.
Volandes et al., 2012, U.S.	Patients with advanced cancer ($n=80$)	Pre-post	Educational video ($n=80$)	Reflexive	Patients viewed an educational video.	Changes in goals of care preference; knowledge & consistency of preferences with code status.	Before viewing video, 10 patients (13%) preferred life-prolonging care, 24 patients (30%) preferred basic care, 29 patients (36%) preferred comfort care, 17 patients (21%) were unsure. Preferences did not change after video, when 9 patients (11%) chose life-prolonging care, 28 patients (35%) chose basic care, 29 patients (36%) chose comfort care, 14 patients (18%) were unsure ($P=0.28$). Compared with baseline, after the video presentation more patients did not want CPR (71% v 62%; $P=0.03$) or ventilation (80% v 67%; $P=0.008$). Knowledge about goals of care & likelihood of resuscitation increased after the video ($P<0.001$). Of patients who did not want CPR or ventilation after video, only 4 patients (5%) had documented DNR order in their medical record (kappa statistic, –0.01; 95% CI, –0.06–0.04).
Green, Levi, 2011, U.S.	2nd year medical students ($n=121$) & patients with cancer ($n=121$)	Pre-post	Computer-based multimedia decision aid designed to help patients w ACP ($n=60$)	Standard AD ($n=61$)	Medical students used either computer-based ACP or standard AD form to introduce ACP to patients & help patients explore their values, priorities, & understanding of common EOL conditions & medical interventions.	Patient satisfaction w ACP methods.	Patients in intervention arm were more satisfied w the computer-based ACP method (8.1 v 6.6, $p<0.01$, where 1 = not at all satisfied, 10 = extremely satisfied).

(*continued*)

Supplementary Table S3. (Continued)

Author, year, and country of origin	*Population*	*Study design*	*Intervention*	*Comparison*	*Methods*	*Outcomes*	*Results*
Uitdehaag et al., 2011, The Netherlands	Patients w new diagnosis of incurable esophageal or head & neck cancer ($n=17$)	Pre-post	Audiorecording (CD) of diagnostic consultation ($n=10$)	No CD ($n=7$)	Intervention received CD recording of consultation, delivery of diagnosis, & discussion of palliative care.	QOL; communication.	Three-quarters of patients appreciated receiving CD; 8/10 patients & 10/10 others listened to CD. No significant differences between groups at baseline or follow-up. Trend towards a poorer QOL but an improved openness to discuss cancer-related issues in CD group.
Yun et al., 2011, Korea	Caregivers of terminally ill patients w cancer & their patients ($n=444$)	RCT	Decision aid (video & companion workbook) showing how to discuss prognosis w patient ($n=216$)	Decision aid (video & companion workbook) showing how to control cancer pain ($n=228$)	Control arm received Korean version of U.S. Nat'l Cancer Institute DVD on pain mgmt. & book on pain control by Korean Ministry of Health & Welfare; experimental arm received 20 minute DVD produced by researchers & companion workbook providing protocol on informing patients of terminal status.	Decision to communicate; caregivers' decisional conflict; patient satisfaction.	No difference in changes in decision to discuss terminal prognosis between the two groups. Conflict ($P=0.003$), uncertainty ($P=0.019$), & value clarity ($P=0.007$) subscale scores & total DCS score ($P=0.008$) improved from baseline to 1 month significantly more in the experimental arm than in the control arm.<br>Depression improved significantly more in experimental arm than in control arm.
Capewell et al., 2010, U.K.	Palliative care cancer patients ($n=15$)	Interrupted time series	Video (DVD) ($n=15$)	Reflexive	Participants viewed 6 minute DVD of interviews with multidisciplinary palliative care staff focused on aspects of cancer pain & use of strong opioids & received booklet reinforcing info in DVD.	Patient pain scores.	Between V1 & V2, total BPI & PPQ scores improved significantly by 9.6% ($p=0.02$) & 17% ($p=0.04$), respectively, with no further improvements at V3.
El-Jawahri et al., 2010, U.S.	Patients with malignant glioma ($n=50$)	RCT	Video ($n=23$)	Verbal narrative ($n=27$)	Controls heard verbal narrative of goals-of-care options at EOL; intervention heard same narrative, then watched 6 minute video depicting 3 levels of medical care: life prolonging, basic, & comfort care.	1a: Preferences for EOL care.<br>2a: Uncertainty re: decision making; comfort level with the video.	Among controls, 25.9% preferred life-prolonging care, 51.9% basic care, 22.2% comfort care. Among intervention, none preferred life-prolonging care, 4.4% preferred basic care, 91.3% preferred comfort care, 4.4% were uncertain ($P<0.0001$). Mean uncertainty score was higher in video group than in verbal group (13.7 v 11.5, respectively; $P<0.002$). 82.6% of intervention arm reported being very comfortable watching the video.

(*continued*)

Author, year, and country of origin	*Population*	*Study design*	*Intervention*	*Comparison*	*Methods*	*Outcomes*	*Results*
Duggleby et al., 2007, Canada	Terminally ill cancer patients age≥60 years ($n=60$)	Pre-post	Video ($n=30$)	Usual care ($n=30$)	Intervention received LWHP (research-based video & choice of hope activities). Trained RNs instructed patients on completing hope activities.	Patient's hope & QOL scores (McGill Quality of Life Questionnaire).	Patients receiving LWHP had statistically significant higher hope ($U=255$, $P=0.005$) & QOL scores at visit 2 ($U=294.5$, $P=0.027$) than control group.
Nonspecific diagnosis, age≥65							
Volandes et al., 2012, U.S.	Patients age≥65 years admitted to 2 skilled nursing facilities ($n=101$)	RCT	Video ($n=50$)	Verbal narrative ($n=51$)	Control arm listened to verbal narrative; intervention arm watched video describing goals-of-care options.	1a: Patients' preferences for comfort versus other options.<br>2a: Concordance of preferences with documentation in medical record.	Control preferences: comfort, $n=29$ (57%); limited, $n=4$ (8%); life prolonging, $n=17$ (33%); uncertain, $n=1$ (2%). Intervention preferences: comfort, $n=40$ (80%); limited, $n=4$ (8%); life prolonging, $n=6$ (12%). Intervention was associated with greater likelihood of choosing comfort (unadjusted RR, 1.4; 95% CI: 1.1–1.9, $p=0.02$). Among controls choosing comfort, 29% had DNR order (κ statistic 0.18; 95% CI: 0.02–0.37); 33% of intervention subjects choosing comfort had DNR order (κ statistic 0.06; 95% CI: 0.09–0.22).
Volandes et al., 2011, U.S.	Subjects age≥65 years at rural primary care clinic ($n=76$)	RCT	Video decision aid ($n=33$)	Verbal description ($n=43$)	Controls heard verbal description of advanced dementia & goals of care; intervention heard same verbal description & viewed video decision aid.	Preferred goal of care in advanced dementia: life-prolonging care, limited care, or comfort care.	Among controls, 31 (72%) preferred comfort; 5 (12%) chose limited; 7 (16%) chose life prolonging. Among intervention, 30 (91%) preferred comfort; 3 (9%) chose limited; none desired life prolonging ($\chi^2=6.3$, $d??=2$, $p=0.047$). Choice of comfort associated with greater health literacy (unadjusted OR 12.1; 95% CI: 2.4–62.6) & randomization to video (unadjusted OR 3.9; 95% CI: 1.0–15.1).
Hamlet et al., 2010, U.S.	Medicare beneficiaries who need EOL care planning ($n=4742$)	RCT	Telephone counseling program ($n=3112$)	Usual care ($n=1630$)	Telephone-based education & counseling about AD, palliative versus aggressive care, hospice enrollment; facilitation of interactions with physicians & hospice agencies; referrals to hospice when appropriate; caregiver support.	1a: deliberate election of less aggressive care as evidenced by reduced medical spending in the last 6 months of life.<br>2a: increased rate of hospice enrollment; duration of hospice care prior to death.	Average Medicare costs \$1,913 lower for intervention group decedents compared with control group decedents in last 6 months of life ($P=0.05$), for a total savings of \$5.95 million. No significant changes in hospice admissions or mean duration of hospice care.

(*continued*)

Supplementary Table S3. (Continued)

Author, year, and country of origin	*Population*	*Study design*	*Intervention*	*Comparison*	*Methods*	*Outcomes*	*Results*
Matsui, 2010, Japan	Japanese adults age≥65 years ($n=121$)	Pre-post	Video & lecture using handout ($n=55$)	Handout only ($n=57$)	Intervention group received 90 minute educational program (video, lecture, handout, & discussion).	Attitudes towards ADs & preference for life-sustaining treatment by means of artificial nutrition.	Intervention group's positive attitude towards ADs increased ($P=0.024$). Preference for life-sustaining treatment by means of artificial nutrition decreased at follow-up ($P=0.008$). At follow-up, twice as many in intervention group had discussed EOL matters with family members and/or their physician.
Volandes et al., 2009, U.S.	Older people age>65 years living in the community ($n=200$)	RCT	Verbal description with video decision support tool ($n=94$)	Verbal description alone ($n=106$)	Control arm listened to verbal narrative describing advanced dementia; experimental group listened to same verbal narrative followed by watching a 2 minute video depicting patient w advanced dementia.	Preferred goal of care: life-prolonging care (CPR, mechanical ventilation), limited care (admission to hosp., antibiotics, no CPR), or comfort care (tx only to relieve symptoms).	Among verbal-only group, 68 (64%) chose comfort care, 20 (19%) chose limited care, 15 (14%) chose life-prolonging care, & 3 (3%) were uncertain. In the video group, 81 (86%) chose comfort care, 8 (9%) chose limited care, 4 (4%) chose life-prolonging care, & 1 (1%) was uncertain ($X^2=13.0$, df$=3$, $P=0.003$). Participants in video group were more likely to prefer comfort care than those in verbal group (AOR 3.9, 95% CI: 1.8–8.6)
Volandes et al., 2009, U.S.	Community-dwelling subjects age≥65 years & their surrogates ($n=28$; 14 dyads)	RCT	Video decision support tool ($n=16$; 8 dyads)	Verbal description ($n=12$; 6 dyads)	Controls heard verbal narrative describing advanced dementia; intervention heard same verbal narrative followed by viewing 2 minute video decision support tool depicting patient with advanced dementia.	Goals of care chosen by patient; goals of care predicted by surrogate; concordance rate of preferences between patients & surrogates.	Among controls, 3 (50%) preferred comfort care, 1 (17%) chose limited care, 2 (33%) chose life-prolonging care. Among surrogates for controls, only 2 correctly chose what their loved one would want if in a state of advanced dementia, yielding a concordance rate of 33%. Among intervention, all chose comfort care. Among surrogates for intervention patients, all correctly chose what their loved one would want if in a state of advanced dementia, yielding a concordance rate of 100%.

(*continued*)

Supplementary Table S3. (Continued)

Author, year, and country of origin	*Population*	*Study design*	*Intervention*	*Comparison*	*Methods*	*Outcomes*	*Results*
Clarke, et al., 2005, U.S.	Senior citizens in established community organizations ($n=18$ groups, ranging from 48–331 members each) & research sites ($n=10$, ranging from 205–1592 members)	Pre-post	Mailed flyer; home-based AD guide, tailored to states in study (CA, IA, PA); telephone counseling	Reflexive	Community-based outreach via direct mail offered home-based critical care & AD guide & individual phone counseling to 18 groups of seniors.	Completion of AD, with and without telephone counseling.	944 households requested a guide. A relation did exist between signing an AD & receiving telephone counseling, but participants also showed strong response to guide without telephone counseling.
Brown et al., 1999, U.S.	Patients age≥75 years who used the Franklin Medical Office ($n=1247$)	RCT	Videotape, illustrated pamphlet, AD forms, guide to their completion ($n=619$)	Cartoon-illustrated pamphlet, ACP forms & guide to their completion ($n=628$)	Participants were mailed collection of printed materials on ACP. Intervention group also received 20 minute videotape on ADs.	Proportion of subjects who placed AD in medical record for the first time.	Placement rates increased almost identically, from 21.2% to 35.0% in written materials only group & from 18.9% to 32.6% in group receiving videotape (95% CI for difference −0.04, 0.04, $p=0.952$).
Yamada et al., 1999, U.S.	Veterans age≥70 years deemed able to make medical care decisions ($n=117$)	RCT	Videotape & two handouts ($n=62$)	One handout ($n=55$)	Controls received handout explaining ADs but not CPR. Intervention received same handout, plus handout on CPR & its outcomes, & viewed 10 minute videotape about ADs.	Knowledge, attitudes, & activity toward ADs & life-sustaining treatments.	At baseline, 27.8% of participants claimed knowledge of AD. Both groups increased knowledge of AD (87.2% intervention, 52.5% control), but stated knowledge of AD was higher in intervention (OR=6.18, $p<0.001$). At baseline, 15% of subjects correctly estimated likelihood of survival after CPR. Intervention increased accuracy (OR=4.27, $p=0.004$), but increase did not persist at follow-up. At conclusion of study, few subjects had discussed CPR or ADs with their physician (OR=0.97, p=NS).
Nonspecific diagnosis, other							
Kannan, Kamalini, 2013, India	Palliative care outpatients ($n=60$)	Interrupted time series	SMS text messaging	Historical data	Patients were trained to send SMS to mobile number provided. Messages were sent on 1st, 2nd, 3rd, 7th, & 14th days of treatment. If patient reply indicated drowsiness, patient was contacted by telephone.	Usability & accuracy of mobile phone as communication tool betw patients & MDs; ability to provide care remotely v require hospital admission.	93% of patients were prompt in response to SMS text prompts; random verification found 100% accuracy of patient responses. Of 60 patients, 22 were admitted for dose titration; all others remained outpatients.
Sudore et al., 2013, U.S.	Racially & ethnically diverse, low-income adults age≥60 years ($n=43$)	Pre-post	Prototype website ($n=43$)	Reflexive	Web-based guide trained patients to identify life goals & preferences for medical care & to communicate these preferences to surrogate decision makers & physicians.	Website efficacy to engage older adults in ACP.	Behavior Change Process Measures average Likert scores increased from 3.1 (0.9) to 3.7 (0.7), $P<0.001$. Action Measures did not change significantly. Precontemplation significantly decreased for most actions (e.g., talking to doctor about desired medical care, 61% to 35%, $P<0.003$), with a mean decrease of 21% (range, 16%–33%).

(*continued*)

Author, year, and country of origin	*Population*	*Study design*	*Intervention*	*Comparison*	*Methods*	*Outcomes*	*Results*
Takahashi et al., 2012, U.S.	Patients age > 60 years with chronic health problems at high risk of hospitalization & ED visits ($n=205$)	RCT	Telemonitoring ($n=102$)	Usual care ($n=103$)	Patients in telemonitoring group relayed biometric & clinical info asynchronously to RN, who reviewed info daily. When required, medical care team would communicate with patient via phone or videoconferencing.	1a: Hospice enrollment.<br>2a: Mean number of days in hospice.	9 patients (9.6%) in telemonitoring group were enrolled in hospice care; 4 patients (4.0%) in usual care group were enrolled ($P=0.12$). Mean number of days in hospice was 57.9 (SD ±99.2) for telemonitoring group, 119.3 (SD ±123.8) for usual care group ($P=0.36$). No significant difference regarding time to hospice referral.
Deep, et al., 2010, U.S.	Patients age > 40 years scheduled to see general internist ($n=120$).	Pre-post	Video	Verbal description	Participants were read verbal description of advanced dementia & asked their preferences for future care (life prolonging, limited, comfort) & rationale for that choice. Then participants watched video of patient w advanced dementia & again stated preferred level of care & rationale.	Impact of video images of a patient on rationale that informs participants' decisions about future care.	Pre-video, postverbal description: 25 chose life-prolonging care, 22 chose limited care, 60 chose comfort care, 13 were uncertain.<br>Post-video: 10 chose life-prolonging care, 10 chose limited care, 107 chose comfort care, none remained uncertain.<br>Video had major impact on participants' decision by imparting knowledge more effectively than words.
Kersholt et al., 2009, The Netherlands	Members of choirs & music associations ($n=183$)	Pre-post	Direct assessment ($n=66$), text ($n=63$), or video ($n=52$)	Within-subject evaluation of options (home, hospice, nursing home); between-subjects method of preference elicitation (direct assessment, text, or video)	Participants randomly assigned to an experimental condition (direct assessment, text, or video) to elicit preferences re: location of death.	Attractiveness of locations of death: home, hospice, nursing home.<br>Confidence in pre/posttest evaluation. Weight of attributes: privacy/hominess; availability of professional care; burden to family. Agreement of text or video w subject's opinion. Subjective evaluation of elicitation method.	With direct assessment, no change in participant's evaluation of 3 places of dying. With text stories, participants became more negative re: home option (66.36 v 72.65), $F(1.63)=6.48$, $P=0.013$, & more positive re: hospice option (49.08 v 41.86), $F(1,63)=7.98$, $P=0.006$. No difference for nursing home option $F(1,63)<1$. With video, participants also became more negative about home option (62.70 v 74.72). $F(1,52)=26.36$, $P \leq 0.0001$, and more negative about hospice (60.29 v. 52.87), $F(1,52)=8.63$, $P=0.005$. Video group also became more positive about nursing home (24.94 v 20.89), $F(1,52)=5.47$, $P=0.023$. Participants changed opinion with stories and videos, and effect was stronger with videos.

(*continued*)

Supplementary Table S3. (Continued)

Author, year, and country of origin	*Population*	*Study design*	*Intervention*	*Comparison*	*Methods*	*Outcomes*	*Results*
Volandes et al., 2009, U.S.	Adult patients age ≥ 40 years scheduled to see a general internist ($n = 146$)	Pre-post	Video decision aid ($n = 146$)	Reflexive	Subjects were asked about preferences for EOL care after hearing verbal description of advanced dementia & were asked to rate level of uncertainty. Subjects then viewed 2 minute video of patient depicting salient features of advanced dementia & were asked again about preferences & uncertainty.	Patient preferences for EOL care & patients' level of uncertainty about EOL care preferences.	Prior to video, average uncertainty scores for subjects with low, marginal, & adequate health literacy were 10.8, 12.4, & 13.5, respectively ($P < 0.0001$). After video, the 3 groups had similar uncertainty about their decisions. Average uncertainty scores for subjects with low, marginal, and adequate health literacy were 13.6, 14.1, and 14.5, respectively ($P = 0.046$).
Schofield et al., 2008, Australia	Patients scheduled to receive their first ever chemotherapy treatment ($n = 100$)	Pre-post	Video (DVD) ($n = 50$)	Usual care (historical controls) ($n = 50$)	Intervention group received usual care plus educational DVD on preparing for chemotherapy & self-management of 8 common side effects.	Patient self-efficacy, anxiety, supportive care needs.	Significant differences found between usual care & intervention groups: for self-perceived curative patients in relation to self-efficacy for seeking social support ($p = 0.044$), with increased confidence in those watching the DVD, and for self-perceived palliative patients in relation to their satisfaction with information about side effects ($p = 0.026$), with increased satisfaction in those watching the DVD. Overall, significant differences were found between self-perceived curative v palliative patients on measures of self-efficacy and supportive care needs, with self-perceived curative patients reporting more confidence and fewer needs.

(*continued*)

Supplementary Table S3. (Continued)

Author, year, and country of origin	*Population*	*Study design*	*Intervention*	*Comparison*	*Methods*	*Outcomes*	*Results*
Volandes et al., 2008, U.S.	White and African American patients presenting to their primary care doctors ($n=144$)	Pre-post	Video decision support tool to help patients overcome low health literacy barriers ($n=144$)	Reflexive	Subjects were asked their preferences for EOL care after hearing verbal description of advanced dementia. Subjects then viewed 2 minute video depicting patient with salient features of advanced dementia & were asked again about their preferences.	Patient preferences for end-of-life care, dichotomized into comfort care and aggressive care.	In unadjusted analyses, African Americans were more likely than whites to have preferences for aggressive care after verbal description (OR 4.8, 95% CI: 2.1–10.9). Subjects with low or marginal health literacy more likely than subjects with adequate health literacy to prefer aggressive care after verbal description (OR 17.3, 95% CI: 6.0–49.9 and OR 11.3, 95% CI: 4.2–30.8, respectively). In adjusted analyses, health literacy (low health literacy: OR 7.1, 95% CI: 2.1–24.2; marginal health literacy OR 5.1, 95% CI: 1.6–16.3) but not race (OR 1.1, 95% CI: 0.3–3.2) was an independent predictor of preferences after verbal description. After watching video of advanced dementia, no significant differences in the distribution of preferences by race or health literacy.
Volandes et al., 2008, U.S.	Spanish-speaking subjects presenting to their primary care doctors ($n=104$)	Pre-post	Video decision support tool ($n=104$)	Reflexive	Patients were asked their preferences for EOL care, then viewed 2 minute video depicting patient with salient features of advanced dementia & were asked again about preferences.	Patient preferences for end-of-life care.	Before seeing video, 42 (40%) subjects preferred comfort care; 43 (41%) desired life-prolonging care; 11 (11%) chose limited care; 8 (8%) were unsure of preferences. Subject preferences changed significantly after video: 78 (75%) of subjects chose comfort care; 8 (8%) desired life-prolonging care; 14 (13%) chose limited care; 4 (4%) were unsure of preferences ($p<0.001$). Unadjusted & adjusted analyses revealed statistically significant difference regarding pre-video preferences based on educational level. After video, differences in preferences based on educational level disappeared.

(*continued*)

Author, year, and country of origin	*Population*	*Study design*	*Intervention*	*Comparison*	*Methods*	*Outcomes*	*Results*
Penrod et al., 2007, U.S.	Patients at 5 acute care & 3 nursing home sites ($n=3557$)	Pre-post	Web-based palliative care report card	Historical data	Interdisciplinary teams established & trained at intervention sites. Web-based palliative care report card implemented on network intranet.	Number of PC consults; percent of inpatient deaths with PC consultation; average # days betw initial PC consultation & deat.h	Number of patients who received PC consults increased by >50%. Percentage of deaths w PC consultation prior to death increased from 23% at baseline to 57%. Average days betw initial PC consultation & patient death increased from 23 days to 106 days.
Volandes et al., 2007, U.S.	Patients age >40 years scheduled to see a general internist at any of 7 study sites ($n=120$)	Pre-post	Video decision support tool ($n=120$)	Reflexive	Comparison of effects of video depiction with verbal description of patient with advanced dementia for individuals selecting level of medical care.	1a: Change in level of care selected.<br>2a: Change in preferences stratified by SES; perceived value of video for understanding, comfort; usefulness of video generally for other diseases.	Intervention significantly increased patient preferences for comfort care as compared with other levels of care.<br>Before video, 60 (50.0%) subjects chose comfort care, 25 (20.8%) chose life-prolonging care, 22 (18.3%) chose limited care, 13 (10.8%) were unsure of preferences. Preferences changed significantly after the video: 107 (89.2%) of subjects chose comfort care, none chose life-prolonging care, 10 (8.3%) chose limited care, and 3 (2.5%) were unsure of preferences ($P<0.001$). Unadjusted analysis revealed statistically significant difference regarding preferences, based on race/ethnicity, before video: 40% of African Americans & 43% of Latinos chose comfort care, compared with 58% of whites ($P=0.04$). Differences also noted for educational level ($P=0.03$). After video, differences in preferences based on race/ethnicity & educational level disappeared.

(*continued*)

Supplementary Table S3. (Continued)

Author, year, and country of origin	*Population*	*Study design*	*Intervention*	*Comparison*	*Methods*	*Outcomes*	*Results*
Brumley, et al., 2006, Australia	All adult patients admitted to domiciliary palliative hospice care (n=NR)	Pre-post	One-page information sheet (in MS Word) updated daily for each patient on computers, faxed to GPs, & downloaded to nurses' PalmPilots	Reflexive	Each day, every patient's purpose-designed single-page information sheet was updated on community palliative care service computers. Patients considered unstable had info sheets faxed to GPs on call.	Number of accurate predictions of unstable palliative care patients resulting in after-hours calls; quality of access to clinical information for nurses & doctors providing after-hours community palliative care.	Nurses reported improved outcomes for patients due to nurses' improved confidence based on increased knowledge of patients' history, current condition & treatments, & plans for future care.
Hanks et al., 2002, U.K.	New inpatient referrals to the PCT (n=261)	RCT	Multidisciplinary specialist PCT (n=175)	Limited telephone advice (n=86)	Full package of advice & support provided by multidisciplinary specialist PCT compared with limited telephone advice.	Physical symptoms & HRQoL; patient, family, & PCP reported satisfaction with care; health service resource use.	Statistically significant improvements in symptoms, HRQoL, mood, & emotional bother among intervention group at 1 week, maintained over the 4 week follow-up ($p<0.001$). Smaller effect seen in controls; no significant differences between groups. Satisfaction with care in both groups was high, no significant difference between them.
Gammaitoni et al., 2000, U.S.	Patients enrolled at university pain clinic (n=74)	RCT	Telephone-based pharmaceutical care program (n=38)	Usual care (n=36)	Palliative care pharmacy company provided telephone-based specialized prescription services tailored to needs of pain medicine clinical practice. Pharmacist remotely monitored patient pharmacotherapy for potential or actual drug related problems.	Delivery of medication; QOL; overall satisfaction with pain clinic program.	Intervention patients perceived that they had better access to medication, more efficient processing of prescriptions, and fewer stigmatizing experiences. They also endorsed pharmacists' behavioral interventions such as medication counseling, availability to answer medication-related questions, and nonjudgmental attitudes when managing opioid prescriptions.
Ho, et al., 2000, Canada	Patients with HIV/AIDS (n=140)	Prospective cohort study	AD documents, educational video, & 3 individual face-to-face counseling sessions	Reflexive	At first interview, participants screened for eligibility, received info re: study, provided consent, completed self-administered AD-PSQ, viewed 17 minute educational video, & received an AD form to complete at home.<br>At second interview, research asst reviewed AD, answered q's, & explained requirements of legally valid AD.<br>At third interview, AD-PSQ repeated, completion of ADs documented by self-report, & ADs photocopied to review for legal validity.	1a: AD completion rate by self-report over ~6 months & examination of AD documents to determine legal validity.<br>2a: Effect on patient satisfaction.	ACP intervention associated w increase in AD completion rates from 16.4% to 40.7% ($p=0.001$), but 23.1% of ADs as completed were legally invalid. Trend toward decreased patient satisfaction w health care ($p=0.07$).

[a]See Supplementary Table S4 for a complete explanation of evidence table codes.
AD, advance directive; CHESS, Comprehensive Health Enhancement Support System; CPR, cardiopulmonary resuscitation; EPP, electronic prompt; NR, none reported; PCT, palliative care team; QOL, quality of life.
