# Supplementary material for: Death in the Digital Age: A Systematic Review of Information and Communication Technologies in End-of-Life Care
Source: J Palliat Med. 2016 Apr 1;19(4):408–20. doi: 10.1089/jpm.2015.0341 (PMC4827321; doi:10.1089/jpm.2015.0341)
Supplement: Supplemental data [file Supp_Table4.pdf]

Supplementary Table S4. Evidence Table Codes

AD = Advance Directive
AD-PSQ = Advance Directive Patient Satisfaction Questionnaire
BPI = Brief Pain Inventory
CAGE = Substance Abuse Screening Tool
CQOL-C = Caregiver Quality of Life Index - Cancer
CSQ = Coping Strategies Questionnaire
DCS = Decisional Conflict Scale
DRS = Decision Regret Scale
ECOG = Eastern Cooperative Oncology Group
EORTC QLQ-C30 = European Organization for Research and Treatment of Cancer Quality of Life Questionnaire for Cancer Patients
ESAS = Edmonton Symptom Assessment System
FAMCARE = Family Care Scale
FPQ = Family Pain Questionnaire
HADS = Hospital Anxiety and Depression Scale
HHI = Herth Hope Index
HRQoL = Health-Related Quality of Life
KPS = Karnofsky Performance Status
MAQ = Morisky's Medication Adherence Questionnaire
MMSE = Folstein Minimental State Examination
MPAC = Memorial Pain Assessment Card
PC = Palliative Care
PCT = Palliative Care Team
PhPI = Pharmacotherapeutic Pain Inventory
POS = Palliative Care Outcome Scale
PPQ = Patient Pain Questionnaire
PPS = Palliative Performance Scale
REALM = Rapid Estimate of Adult Literacy
RSCL = Rotterdam Symptom Checklist
SERVQUAL = Quality of Service Scale
THQ = Treatment Helpfulness Questionnaire
VAS = Visual Analogue Scales
VISN = Veteran Integrated Service Networks
