# Supplementary material for: Death in the Digital Age: A Systematic Review of Information and Communication Technologies in End-of-Life Care
Source: J Palliat Med. 2016 Apr 1;19(4):408–20. doi: 10.1089/jpm.2015.0341 (PMC4827321; doi:10.1089/jpm.2015.0341)
Supplement: Supplemental data [file Supp_Table5.pdf]

Supplementary Table S5. Web Resources

Primary author	*Website name*	*url*
Web resources from included studies		
Green and Levi	Making Your Wishes Known	www.makingyourwishesknown.com/
Gustafson et al.	Comprehensive Health Enhancement Support System (CHESS™)	www.chess.wisc.edu/chess/projects/about_chess.aspx
Sudore et al.	Prepare	prepareforyourcare.org/
Volandes et al.	ACP Decisions	www.acpdecisions.org/about/
Additional web resources		
Back et al.	VitalTalk	www.vitaltalk.org/
Goodman et al.	The Conversation Project	theconversationproject.org/
Hebb et al.	Let's Have Dinner and Talk About Death	deathoverdinner.org/
Towey et al.	Aging with Dignity: Five Wishes	agingwithdignity.org/five-wishes/about-five-wishes
